# *In Situ* Oxygen Dynamics in Coral-Algal Interactions

**DOI:** 10.1371/journal.pone.0031192

**Published:** 2012-02-02

**Authors:** Daniel Wangpraseurt, Miriam Weber, Hans Røy, Lubos Polerecky, Dirk de Beer, Suharsono, Maggy M. Nugues

**Affiliations:** 1 Coral Reef Ecology Group (CORE), Department of Ecology, Leibniz Center for Tropical Marine Ecology, Bremen, Germany; 2 Max Planck Institute for Marine Microbiology, Bremen, Germany; 3 HYDRA Institute for Marine Sciences, Elba Field Station, Campo nell'Elba, Italy; 4 Department of Biological Sciences, Center for Geomicrobiology, Aarhus University, Aarhus, Denmark; 5 Research Center for Oceanography, Indonesian Institute of Sciences, Jakarta, Indonesia; University of Canterbury, New Zealand

## Abstract

**Background:**

Coral reefs degrade globally at an alarming rate, with benthic algae often replacing corals. However, the extent to which benthic algae contribute to coral mortality, and the potential mechanisms involved, remain disputed. Recent laboratory studies suggested that algae kill corals by inducing hypoxia on the coral surface, through stimulated microbial respiration.

**Methods/Findings:**

We examined the main premise of this hypothesis by measuring *in situ* oxygen microenvironments at the contact interface between the massive coral *Porites* spp. and turf algae, and between *Porites* spp. and crustose coralline algae (CCA). Oxygen levels at the interface were similar to healthy coral tissue and ranged between 300–400 µM during the day. At night, the interface was hypoxic (∼70 µM) in coral-turf interactions and close to anoxic (∼2 µM) in coral-CCA interactions, but these values were not significantly different from healthy tissue. The diffusive boundary layer (DBL) was about three times thicker at the interface than above healthy tissue, due to a depression in the local topography. A numerical model, developed to analyze the oxygen profiles above the irregular interface, revealed strongly reduced net photosynthesis and dark respiration rates at the coral-algal interface compared to unaffected tissue during the day and at night, respectively.

**Conclusions/Significance:**

Our results showed that hypoxia was not a consistent feature in the microenvironment of the coral-algal interface under *in situ* conditions. Therefore, hypoxia alone is unlikely to be the cause of coral mortality. Due to the modified topography, the interaction zone is distinguished by a thicker diffusive boundary layer, which limits the local metabolic activity and likely promotes accumulation of potentially harmful metabolic products (e.g., allelochemicals and protons). Our study highlights the importance of mass transfer phenomena and the need for direct *in situ* measurements of microenvironmental conditions in studies on coral stress.

## Introduction

In the last three decades, coral reefs have been exposed to an increasing intensity and frequency of human stressors. We are now seeing an unprecedented decline of coral reefs worldwide, owing primarily to the combined effects of overfishing, pollution, rising sea surface temperatures, ocean acidification and emerging coral diseases [1]–[4]. Degradation of coral reefs typically involves a shift in community structure from a coral-dominated reef to an algal-dominated reef, a process known as ‘coral-algal phase shift’ [5]–[7]. Despite being a well-documented phenomenon, the underlying dynamics and mechanistic processes leading to algal dominance are still unclear. It remains disputed whether algae acquire space by colonizing open substrates after a coral has died, or by actively overgrowing and out-competing neighboring corals [8]. Studies addressing coral-algal competition have produced variable results, leaving much to be learned about the properties and mechanisms that determine the winners and losers of this battle [9]–[12].

Benthic algae can compete with corals through a number of physical and chemical mechanisms. Negative effects of algae on coral health have been attributed to direct physical effects such as shading, abrasion or smothering [10], [13], [14]. Increasingly, the importance of chemically- and microbially-mediated mechanisms is being recognized. Algae can exude primary or secondary metabolites that are toxic to corals and/or coral-associated microorganisms [15]–[18], or they can act as a reservoir for microbial pathogens [19]. In a small-scale laboratory experiment, Smith et al. [20] showed that, when corals and algae were placed in chambers together, but separated by a fine filter to prevent exchange of particulate matter, the coral fragments suffered 100% mortality. Microsensor measurements on the surface of the coral tissue adjacent to algae revealed that these areas were hypoxic. Addition of antibiotics to the water bath prevented both the deleterious effects to the corals and the hypoxia. It was concluded that algae exude primary metabolites (i.e. sugars and carbohydrates), which enhanced microbial respiration and subsequently led to localized hypoxia and coral mortality. Building upon this, a recent laboratory-based study described the occurrence of hypoxia at the boundaries between corals and some turf or fleshy macroalgae. In contrast, supersaturated oxygen levels were found at the contact zone with CCA [21]. It was thought that hypoxia is a general phenomenon in interactions with macroalgae, representing a constant source of stress to corals. However, these laboratory observations have yet to be confirmed *in situ*.

The oxygen microenvironment of healthy corals is a dynamic microenvironment primarily regulated by diffusive exchange of oxygen through the DBL [22] and light-dependent metabolic activity of the coral [23], [24]. During day-time, oxygen production by the corals' symbiotic zooxanthellae can lead to supersaturation on the coral surface, while at night-time, coral community respiration can induce hypoxia. The function of the DBL in controlling the oxygen concentration at the coral surface is well documented (e.g., [23], [25]). For instance, oxygen values on coral tissue of *Favia* sp. were reduced from about 60% air saturation at a flow velocity of 5 cm s^−1^ to anoxia under stagnant water [23]. Given these extreme diel and flow-dependent variations in oxygen microhabitats on healthy corals, it is critical to ask whether detrimental oxygen conditions at the coral-algal interface prevail *in situ* under a natural regime of flow, water exchange and light.

Here, we examined the spatial competition between corals and two common algal groups, turf algae and CCA, from coral reefs in Derawan Island, Indonesia. We focused on communities of turf algae (mixed assemblages of filamentous algal and cyanobacterial species with an average height <10 mm) [26] and CCA that interacted with the Indo-Pacific reef building massive coral *Porites*. Turf algae have become one of the most abundant components on modern day reefs worldwide and compete with corals on degraded nutrient rich reefs [27]. CCA have positive roles for the maintenance of coral-dominated reef communities as they contribute to limestone formation and are important settlement inducers of coral larvae [28]. They have also been found to prevent the recruitment and growth of the macroalgae *Ulva fasciata* [29]. The specific goals of this study were therefore to 1) quantify the abundance of *Porites*-Turf and *Porites*-CCA interactions, 2) follow interaction borders over time to assess the rates of coral overgrowth by algae and how this rate varies between algal types and, 3) characterize chemical microenvironments at the coral-algal interface *in situ* with respect to oxygen concentration and exchange.

## Results

### Abundance of Coral-algal Interactions

**Figure 1:**
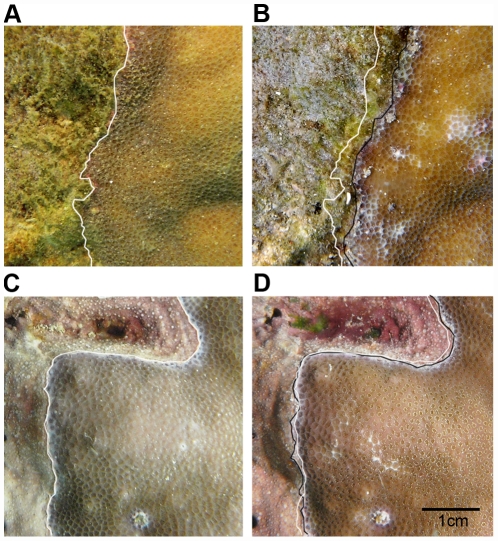
Changes in initial and final contact boundaries between the massive coral *Porites* spp. and algae. A) *Porites* spp. vs. turf algae at day zero and B) after 60 days, C) *Porites* spp. vs. CCA at day zero and D) after 60 days. White lines represent initial coral-algal boundaries and black lines represent final boundaries after 60 days.

We found that massive *Porites* spp. colonies had a mean interaction border of 39 cm (±0.96 SE, n = 39 colonies). This border was predominantly in contact with turfs (28.95 cm±1.02 SE, ∼74%), followed by CCA (7.38 cm±0.57 SE, ∼19%) and ‘other’ benthos including fleshy macroalgae (2.67 cm±0.17 SE, ∼7%). Time-series photomonitoring demonstrated that both turf and CCA overgrew the coral tissue (Figure 1A–D). Turf algae and CCA advanced at rates of 0.58 mm per month (±0.12 SE, n = 5) and 0.11 mm per month (±0.07 SE, n = 4) respectively. This suggested a greater rate of coral overgrowth by turf algae than by CCA, but this difference was not significant (1-way ANOVA: F_1,7_ = 1.82, p = 0.22).

### Oxygen Profiles

**Figure 2:**
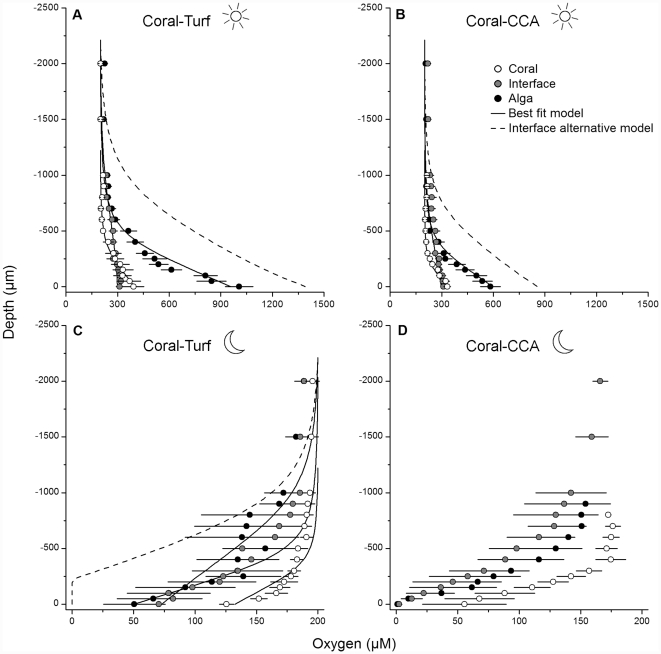
*In situ* oxygen microprofiles in coral-algal interactions during the day and night. Data points are means ± SE. Depth 0 µm refers to the organism surface, and negative values indicate profiling upwards into the water column. Dotted lines show the oxygen distribution at the interface based on the initial assumption of a constant algal and coral flux. Solid lines illustrate the best fit model at each measuring spot (‘Coral’, ‘Interface’ and ‘Alga’). A) *Porites*-turf interactions during the day (n = 7 profiles), B) *Porites*-CCA interactions during the day (n = 9), C) *Porites*-turf interactions during the night (n = 3) and D) *Porites*-CCA interactions during the night (n = 3). Oxygen distribution could not be modeled for these measurements as the flux at the interface was mass transfer-limited.

During day-time (downwelling irradiance of ∼660 µmol photons m^−2^ s^−1^), hypoxia at the coral-algal interface was not found in any of our 16 *in situ* microprofiles. In both interaction types, oxygen surface concentrations differed among the three sampling points (1-way ANOVA; coral-turf: F_2,18_ = 41.14, p<0.001; coral-CCA: F_2,24_ = 17.93, p<0.001). Turf algae had significantly higher oxygen values (1006 µM±81 SE) than both the coral (393 µM±59 SE) and the interface (311 µM±20 SE), which did not differ from each other (Tukey's HSD post hoc test; Figure 2A). Similarly, surface oxygen concentrations at the CCA averaged 583 µM (±58 SE) and were significantly higher than concentrations at the coral and the interface (332 µM±20 SE and 309 µM±10 SE, respectively; Figure 2B).

At night-time, all measured profiles showed values below air-saturation, reflecting oxygen consumption. Oxygen values differed significantly among the three sampling points in coral-turf interactions (F_2,6_ = 6.48, p = 0.03). The coral (126 µM±6 SE) showed significantly higher oxygen values than the turf (50 µM±25 SE), but both did not differ from the interface which had intermediate values (70 µM±5 SE) (Figure 2C). In coral-CCA interactions, oxygen levels at the algae and the interface were very low (1.0 µM±0.6 SE and 2.0 µM±2.44 SE, respectively), while average values at the coral (55 µM±34 SE) were higher, but differences among the three sampling points were not significant (F_2,6_ = 2.46, p = 0.17; Figure 2D). The oxygen microenvironment at the coral-algal interface was thus highly dynamic, ranging from supersaturated values during the day to hypoxic and anoxic values at night.

The DBL differed significantly among the three sampling points in both interaction types (coral-turf: F_2,18_ = 9.28, p = 0.002; coral-CCA: F_2,24_ = 7.04, p = 0.004). The DBL thickness above the coral-turf interface was significantly higher (∼2.5 times) than above the coral and above the turf algae. The DBL above the coral surface and turf algae was equally thick. DBL values at the coral-CCA interface were significantly higher (∼3.1 times) than at the coral itself and nearly two times higher than the values at the CCA although this difference was not significant (Table 1).

**Table 1:** DBL thickness and oxygen flux in interactions between the massive coral *Porites* spp. and algae.

	Coral-turf (n = 7)			Coral-CCA (n = 9)		
	DBL	P_N_	R_D_	DBL	P_N_	R_D_
Coral	353±206	0.15±0.06	0.10±0.16	248±110	0.20±0.08	0.13±0.14
Interface	961±378	nd	nd	770±349	nd	nd
Alga	424±169	0.61±0.28	0.08±0.11	402±170	0.34±0.18	0.10±0.14

DBL thickness (µm), net photosynthesis rate (P_N_) and dark respiration rate (R_D_, both in nmol O_2_ cm^−2^ s^−1^) at the three sampling points (‘Coral’, ‘Interface’ and ‘Alga’) for each coral-algal interaction type (coral-turf and coral-CCA). Data are means ±95% Confidence Intervals. Note that oxygen fluxes at the interface between coral and algae were not calculated directly from the microprofiles due to violated assumptions in the one-dimensional Fick's first law of diffusion. Therefore the flux at the interface was modeled.

The net photosynthesis rate at the turf surface was significantly higher (∼4 times) than the rate at the coral surface (F_1,12_ = 15.36, p = 0.002), while dark respiration rates did not differ (F_1,4_ = 0.18, p = 0.69). Differences between coral and CCA surfaces in both net photosynthesis and dark respiration rates were not significant (P_N_: F_1,16_ = 2.67, p = 0.12, R_D_: F_1,4_ = 0.43, p = 0.55).

### Two-Dimensional Oxygen Distribution at the Coral-Turf Interface

**Figure 3:**
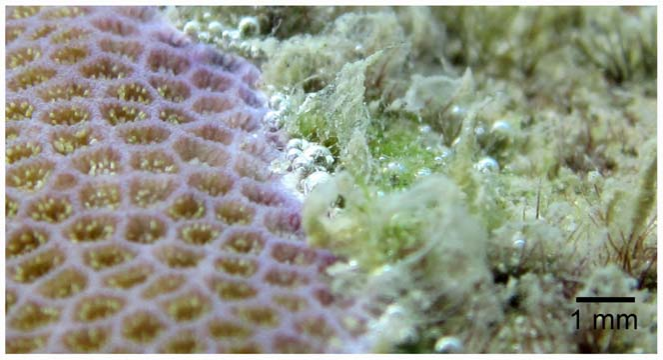
Microtopography of the contact border between the massive coral *Porites* spp. and turf algae. Note the little depression at the contact border, the healthy looking coral surface is slightly elevated above the neighboring algae. The bubbles lining up at the contact border are indicative of a combined effect of reduced diffusive transport due to increased DBL thickness and oxygen supersaturation. The picture was taken at ∼4 m depth.

**Figure 4:**
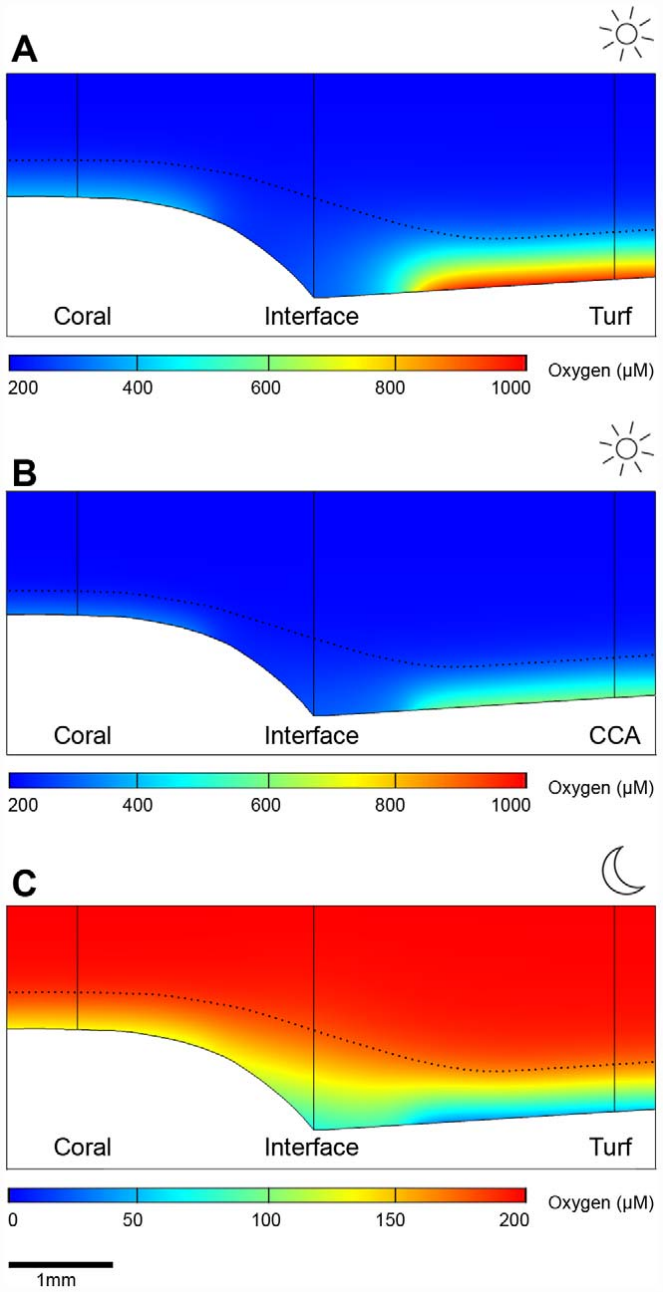
Best match scenarios of oxygen exchange dynamics between the massive coral *Porites* spp. and algae. The two-dimensional oxygen distribution was modeled to best match the measured *in situ* oxygen microprofiles at the three measuring spots (‘Coral’, ‘Interface’ and ‘Turf/CCA’). Dotted black lines illustrate the thickness of the DBL. Sun and moon symbols represent day and night measurements, respectively. Note the different scales in oxygen concentration for the day and night. A) Day-time scenario for coral-turf, B) Day-time scenario for coral-CCA, C) Night-time scenario for coral-turf.

The oxygen flux at the interface between coral and algae could not be directly derived from the microprofiles because of heterogeneous topography (Figure 3). The oxygen flux was therefore modeled. We first assumed that net oxygen production from the coral and turf was constant all the way to the interface. Modeling the oxygen distribution using this assumption produced much higher oxygen concentrations at the coral-algal interface than measured *in situ* (Figures 2A, Figure S1A). To match the *in situ* data, we reduced the oxygen production at the coral-algal interface. Reducing the production rate to zero on either side of the coral-algal interface alone was not sufficient to reproduce the measured data (Figures S1B, S1C). The best fit was found by simulating a 2 mm wide band of zero oxygen flux centered at the competition margin (Figures 2A, 4A). An alternative scenario was that the coral at the interface was net heterotrophic, potentially due to enhanced respiration, while the turf was unaffected. However, it was not possible to match the modeled and measured data in this scenario.

Night-time measurements were modeled in a similar manner. In the first run, we applied the respective night-time flux across the entire coral and the entire turf. The model showed that this flux could not be supported, as the interface would have turned anoxic, which would be in contrast to the oxygen concentrations measured *in situ* (average of 70 µM; Figure 2C). Thus, we conclude that the night-time respiration rate at the interface must be lower than at the coral and at the turf. We matched the model to the data by modifying the flux in the same 2 mm wide band as in the day-light simulation. A flux corresponding to 50% of the night-time coral flux and 33% of the night-time turf flux produced a good match (Figures 2C, 4C).

### Two-Dimensional Oxygen Distribution at the Coral-CCA Interface

The oxygen distribution at the coral-CCA interface was modeled in the same way as for the coral-turf interaction. Again, the model predicted reduced photosynthesis at the interface but to a lesser extent than in the coral-turf case. The best match to the data was found by setting the production rate at the interface to 11% of the rate of healthy coral tissue (Figures 2B, 4B). At night, both the interface and the CCA were anoxic at the surface (Figure 2D). In this situation, the oxygen flux was controlled by the thickness of the DBL. The oxygen demand in the model could therefore be increased indefinitely without influencing the predicted profiles, and it was thus not possible to deduce the oxygen demand by this approach.

## Discussion

Hypoxia was not a consistent feature at the zone of contact between corals and algae *in situ*. In both *Porites*-turf and *Porites*-CCA interactions, the interface was characterized by a dynamic oxygen microenvironment with extreme diel fluctuations, ranging from supersaturated values during the day to hypoxic and anoxic values at night, and did not significantly differ from the coral. Our results, however, cannot be generalized to other types of coral-algal interactions. One species of fleshy macroalgae has been found to transmit pathogens to a coral [19], which may have obvious effects on the physiology near the coral-algal interface. Similarly, corals may have different sensitivities to the presence of algae and hypoxia. In laboratory conditions, the genus *Porites* showed the least amount of mortality in contact with algae [20]. Here, however, coral mortality did occur, as demonstrated by positive rates of overgrowth of massive *Porites* spp. by turf algae and, to a lesser extent, by CCA. Together these results provide limited support for hypoxia alone as the cause of coral mortality in our study. If hypoxia is one of the steps leading to coral death, it needs to be regarded as a fluctuating stressor that affects corals during specific environmental conditions, which is likely at night and/or during periods of reduced flow.

The *in situ* measurements and the numerical model showed reduced net photosynthesis and an increased DBL thickness at the interface between corals and algae. With the concept documented in this study we cannot directly quantify the individual oxygen contribution of each organism belonging to the coral holobiont on its own (i.e. coral host, zooxanthellae and microbes) but only the overall oxygen and mass-transfer conditions of the coral microenvironment. Therefore it is unclear whether the reduced oxygen flux is potentially indicative of somewhat enhanced microbial respiration or rather represents generally reduced metabolic activity (i.e. photosynthesis and respiration) of the coral holobiont. The night-time model suggested reduced dark respiration at the interface. Since a microbial biofilm fed by dissolved organic matter from the algae during day is likely to also have high rates of metabolism during the night, the hypothesis of reduced metabolic activity appears more plausible. Although care must be taken when extrapolating results from the night-time model as it is based on one colony per interaction, consistent results among colonies during day-time suggest that the night-time oxygen data is likely representative of the overall trend for *Porites* spp.-turf and *Porites* spp.-CCA interactions. The reduced metabolic activity at the coral-algae interface could result from the transfer and accumulation of metabolites produced by the corals and algae and/or a greater isolation from essential solutes of the ambient water mass, which are both intensified for thicker DBL.

Benthic algae have been found to exude toxins, resulting in strong negative effects on corals and coral-associated organisms [15]–[18]. Similarly, corals produce a wide range of anti-bacterial [30], [31], anti-fouling [32], anti-fungal [33] and competitor deterrent solutes (e.g., [34], [35]), which are likely to harm neighboring algae. The mutual nature of the competition between corals and algae has been demonstrated previously [9], [12], [36], and agrees well with our best fit model which simulated a 2 mm wide band of reduced oxygen flux centered at the contact border.

**Figure 5:**
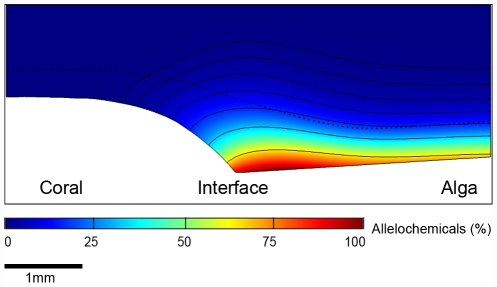
Transport of algal allelochemicals at the coral-algal interface. The figure shows the modeled steady state distribution of a low molecular weight substance released by the algae. The lowermost isoline illustrates 64% of the total concentration of the substance. Subsequent isolines represent a halving of the effect. The dotted black line illustrates the thickness of the DBL, based on measurements in coral-turf interactions. The impact of the alga extends to ∼1 mm over the coral.

The two-dimensional model used to interpret oxygen distribution can be used to estimate the distance across which corals and algae can effectively exchange allelochemicals horizontally in the turbulent reef environment. We assumed an even rate of production of a low molecular weight substance across the surface of the algae-covered substrate, and calculated a steady state distribution that would be reached after a few minutes. The result showed a ‘halo’ of the substance around the algae that was restricted to the DBL and extended only 1 mm across the border to the coral (Figure 5). This agreed well with the 1 mm wide zone of reduced metabolic activity over the coral indicated by the interpretation of the oxygen data and model (Figure 4A–C). This simulation was also consistent with observations documenting the occurrence of chemically mediated effects only at areas of direct contact [17], [37].

Reduced vertical growth at the edge of a coral colony automatically forms a crevice between the coral and the surrounding biogenic rock (Figure 3). The DBL does not follow the surface into such small cracks, which leads to increased DBL thickness at the interface, as indicated by our data. Once a crack is formed, this can lead to a secondary negative effect on the coral due to enhanced transport resistance of solutes and metabolites between the coral and the water column. Previous studies showed that the combination of locally reduced pH and oxygen can rapidly kill coral tissue [38] and that a decrease in ambient pH values facilitated coral overgrowth by benthic algae [39]. During periods of net respiration, the thick DBL will facilitate the development of reduced pH and low oxygen levels by accumulating protons and limiting oxygen supply at the coral surface. It is thus likely that stressful pH and oxygen levels develop temporarily at night. However, future studies are needed to verify whether a decrease in local pH occurs at the competition zone. The thickened DBL increases the exposure of coral margins to algal generated metabolites, highlighting the importance of protecting reefs against the proliferation of chemically damaging seaweeds. The thickness of the DBL also controls coral uptake rates of metabolically important nutrients such as phosphate and ammonium, whereby a thickening of the DBL leads to a decrease in nutrient uptake [40], [41]. Thus, the reduced vitality of the coral edge and the resulting crevice might be self-enhancing, raising the possibility of a negative feedback mechanism in which reduced metabolism of the coral edge promotes crevice formation, which in turn leads to a more detrimental microenvironment and further decrease in coral health.

Coral-CCA and coral-turf interactions did not show strong differences in oxygen concentrations at the interface, although results are not easily comparable since measurements were not done simultaneously. Our model, however, showed a greater reduction in photosynthesis at the interface in coral-turf interactions compared to coral-CCA interactions. This suggested that CCA have less detrimental effects on corals than turf algae which agrees well with the general trend of coral overgrowth observed in this study, as well as in previous investigations (e.g., [27]).

### Conclusions

In search for *in situ* evidence of hypoxia in coral-algal interactions, we found that hypoxia was not a general phenomenon at the interface between the massive coral *Porites* spp. and two major groups of benthic algae (turf and CCA). Enhanced respiration is thus unlikely to be the direct cause of coral tissue mortality. We argue that the coral-algal interface is a zone of low metabolic activity caused by the accumulation of metabolites released by corals and algae. These impacts are facilitated by an enhanced thickness of the DBL, which in turn is attributable to a depression in the local topography. Our findings imply that mass transfer phenomena play an important role in coral-algal competition. Knowledge on the microenvironmental regulation of coral stress will provide a detailed understanding of the mechanisms that trigger coral reef degradation.

## Materials and Methods

### Ethics Statement

The research was approved by the Indonesian State Ministry of Research and Technology (RISTEK) and was performed in strict accordance with Indonesian regulations for field research (research permit number CD4892037).

### Study Site

Field-work was carried out on the reef flat in Derawan Island, east Kalimantan, Indonesia (N 02°16′57″, E 118°14′54″) from January to March 2010. The study area was 20 km offshore from the mainland. It was directly affected by the discharge from the nearby Berau River, which can transport significant sediment loads [42]. Over the past ten years, the reefs of Derawan Island have experienced a decrease in coral cover with subsequent replacement by benthic algae as a result of heavy sedimentation, disease and an outbreak of the corallivorous starfish *Acanthaster planci* [43], [44].

### Coral-Algal Interaction Surveys and Monitoring

The abundance of contact boundaries between massive *Porites* and benthic organisms was determined over eight randomly placed belt transects (2 m width) at depths of 3 to 10 m and orientated parallel to the shoreline. Massive *Porites* colonies were not identified to species level, however it was ensured that all colonies showed the same colony shape and polyp morphology (as observed through a magnifying glass). For the first five colonies encountered per transect, the length of the coral-benthos border(s) was measured to the nearest centimeter on each colony. Benthic organisms were categorized as turf algae, CCA and ‘other’. The ‘other’ category mainly included fleshy macroalgae, ascidians and sponges.

Coral-algal interactions were monitored photographically to follow the temporal changes in position of boundaries between living massive *Porites* spp. tissue and algae. For each of the two interaction types (coral-turf and coral-CCA), five colonies were randomly selected at about 4–7 m depths. All colonies came from a small area (∼300 m^2^) within our study site to minimize macroenvironmental variations (i.e. downwelling irradiance and flow velocity). On each colony, a 10×10 cm quadrat delimited by four stainless-steel nails at each corner, was positioned along the coral-algal boundary and photographed over a 60 day period using a Sea & Sea DX-1G camera with a 24 mm lens and external strobe. Changes in the position of the coral-algal boundary were analyzed in Adobe Photoshop (CS2, Adobe Systems Inc.) using distinctive polyp structures as lines of reference to redraw the final boundary next to the initial boundary. The gain or loss in area of algae was calculated by subtracting the surface area of algal retreat from algal advance and dividing it by the initial length of interaction. One colony with a coral-CCA interaction had to be removed because of coral tissue loss unrelated to algal overgrowth (sand burying).

### Oxygen Microsensor Construction and Calibration

Amperometric Clark-type oxygen microsensors with a guard cathode were constructed according to Revsbech [45]. To minimize the potential of sensor breakage when approaching the coral surface, sensors were built with a thin flexible upper part (ca. 10 cm in length) and a slightly thickened outer case of the sensor tip. Sensors were painted white to increase visibility underwater. Microsensors had a tip diameter of 10–50 µm and a stirring sensitivity of <1.5%. Sensors were linearly calibrated before and after each dive at *in situ* temperature and salinity against air saturated seawater and anoxic sediment. The percent air saturation was transformed to µM oxygen as in Garcia and Gordon [46].

### *In Situ* Oxygen Microsensor Measurements

The characterization of the oxygen microenvironment in the two types of coral-algal interactions was carried out *in situ* during the day and the night using a diver-operated motorized microsensor profiler for underwater field operations (DOMS) [47]. Day-time measurements (9am to 4pm) were performed on three randomly selected interactions from the five photomonitored coral-algal interactions. Oxygen profiles were measured within the monitored area at three points along an axis perpendicular to the coral-algal interface: 1) apparently healthy tissue 4±1 cm away from the interface (‘Coral’), 2) the coral-algal interface (‘Interface’) and, 3) apparently healthy algae 4±1 cm away from the interface (‘Turf’ or ‘CCA’). Oxygen profiles were measured at the three points in a random sequence to reduce any bias related to light and flow conditions, and the procedure was repeated three times at randomly selected locations within the monitored area. Measurements on coral tissue were exclusively conducted on the coenosarc (tissue between polyps) in order to minimize the influence of tissue movement [23] and the spatial heterogeneity of coral photosynthesis [48], [49].

For each oxygen profile, the microsensor was carefully positioned at an angle of approximately 20° from the surface by looking under a magnifying glass and manually moving the micromanipulator of the DOMS until a minute bent of the sensor was observed. The oxygen signal was allowed to stabilize before profiles were measured upwards into the overlying water column in 50–500 µm steps by the DOMS. The sensor was allowed 20 seconds resting time between each measurement, and each measurement consisted of an average of 10 data-points collected over 10 seconds.

Night-time measurements (7–10 pm) were conducted on one randomly selected colony per interaction type using the same measuring approach as for day-time measurements. Artificial illumination is required to determine the surface position at night, which leads to oxygen production by photosynthesis. The oxygen reading was therefore allowed to reach a steady state at darkness before profiling started. This occurred within 1 to 5 min after the light source was switched off.

During all measurements, net current flow velocity in the overlying water 1–2 m from the measured coral colony averaged 8.9 cm s^−1^ (±2.1 SD), as determined by tracing small particles in the water column. Average downwelling irradiance during daytime measurements was 663 µmol photons m^−2^ s^−1^ (±183 SD), measured next to the coral colony with a cosine corrected quantum sensor (Li-192) connected to a data logger (Li 1400, Li-Cor, USA).

### Oxygen Data Analyses and Modeling

For all analyses, it was assumed that oxygen profiles were measured in steady state. We aimed at taking measurements on sunny days, with a calm sea and between tides to minimize environmental variations. Three consecutive profiles from two measuring days (total of 6 profiles) had to be discarded due to cloudy weather. For the remaining profiles the effective DBL thickness was calculated from the intercept between the linear extrapolation of the oxygen profile at the coral surface and the bulk concentration [22]. For ‘Coral’, ‘Turf’ and ‘CCA’ sampling points, areal rates of net photosynthesis and dark respiration were calculated from oxygen profiles in the light and dark, respectively, using Fick's first law of diffusion with a diffusion coefficient of 2.48×10^−5^ cm^2^ s^−1^ (calculated for *in situ* temperatures of 29°C and salinity of 35‰) [50].

Calculation of diffusive fluxes from the simple Fick's first law of diffusion is only valid for a flat surface without substantial heterogeneity, such as the coral or algal surfaces [23], [51]. These requirements are, however, not fulfilled at the coral-algal interface. To approach this problem, we deduced the topography at the interface based on the measured DBL thicknesses and the way the DBL follows the local topography as a smoothed out blanket [52], [53]. A thick DBL measured at the interface was ascribed to a depression in the local topography. This model assumption is reasonable since dead coral skeleton is exposed to bioerosion [54] and benthic algae can reduce coral growth (skeletal extension) and tissue thickness in their vicinity [13], [55], resulting in healthy coral tissue being topographically elevated. This topography was confirmed as a common phenomenon from images from the reef (Figure 3).

The sketched topography was imported into the finite element modeling software Comsol Multiphysics, which can calculate diffusion fields in complex geometries. We modeled concentration and transport from the surface and 3 mm into the water column. The molecular diffusion coefficient (D_m_) was set to 2.48×10^−5^ cm^2^ s^−1^ (see above). Eddy diffusion (D_e_) was modeled such that D_e_ is proportional to the distance from the surface raised to the 4^th^ power, and such that D_e_ = D_m_ at the upper boundary of the effective DBL: De = Dm x (z^4^/Z_eff_^4^) where z is the distance above the surface and Z_eff_ is the thickness of the effective diffusive boundary layer. The left and right boundaries were set to symmetry and the upper boundary towards the water column to 200 µM oxygen. The oxygen flux to the lower boundary (i.e., the coral or algal surface) was adjusted until the modeled oxygen distribution matched the measured microprofiles.

Differences in oxygen surface concentrations and DBL among the three sampling points (coral, interface and algae) were tested using 1-way ANOVA followed by Tukey's HSD post hoc test. Differences in photosynthesis and respiration were only tested between coral and algae since they could not be calculated directly from the microprofiles at the interface. Data were log(x+1) transformed if necessary.

## Supporting Information

Figure S1 **Simulated scenarios of oxygen exchange dynamics between the massive coral** ***Porites*** **spp. and turf algae.** A) Scenario 1: The flux of coral and turf is constant towards the interaction zone. B) Scenario 2: The flux of coral is zero, but constant for turf. C) Scenario 3: The flux of turf is zero, but constant for coral. None of these scenarios matched the *in situ* data.
(TIF)
