# Supplementary figures and images for: In Situ Oxygen Dynamics in Coral-Algal Interactions
Source: PLoS One. 2012 Feb 2;7(2):e31192. doi: 10.1371/journal.pone.0031192 (PMC3271105; doi:10.1371/journal.pone.0031192)

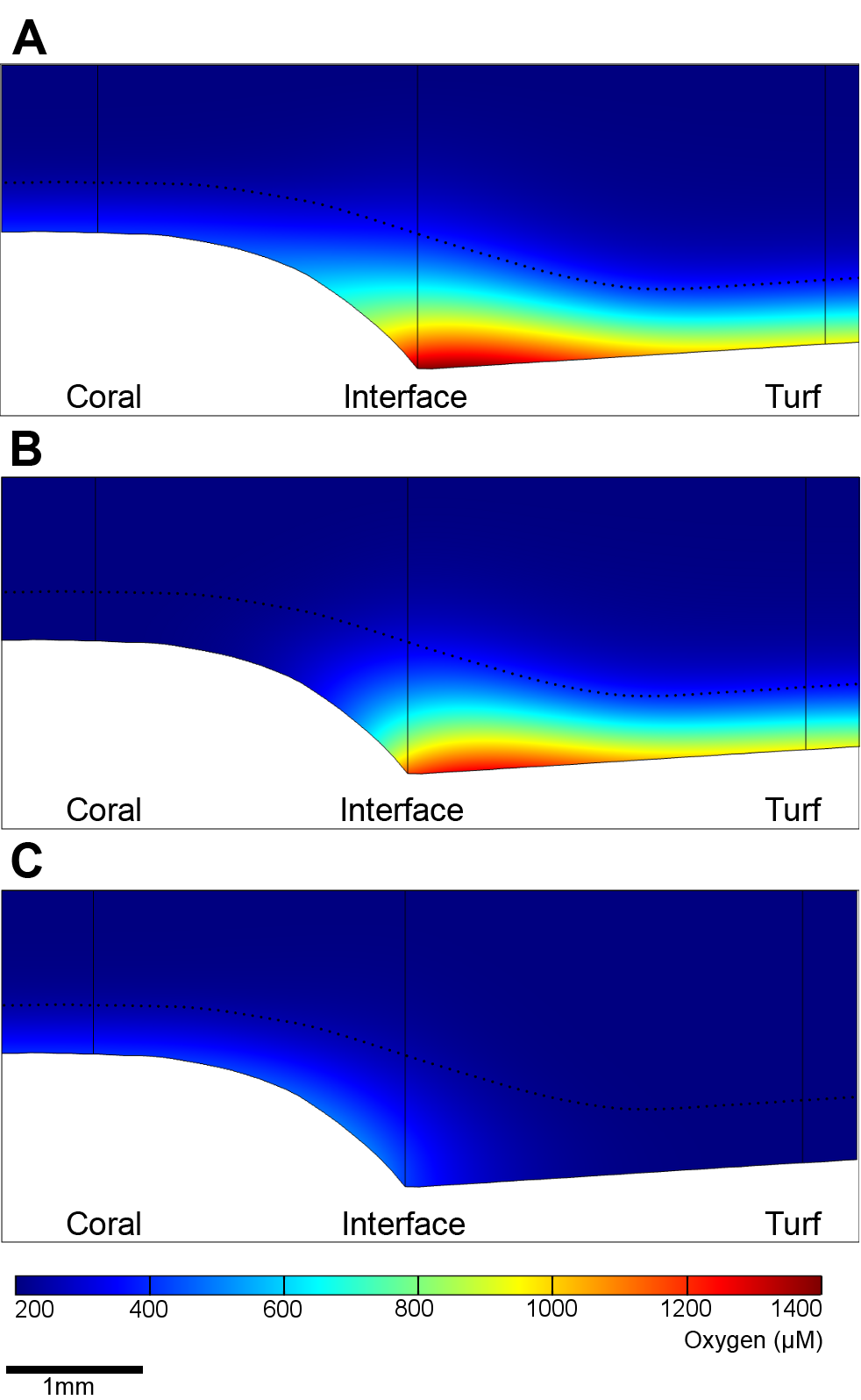

Supplement: Figure S1 — Simulated scenarios of oxygen exchange dynamics between the massive coral Porites spp. and turf algae. A) Scenario 1: The flux of coral and turf is constant towards the interaction zone. B) Scenario 2: The flux of coral is zero, but constant for turf. C) Scenario 3: The flux of turf is zero, but constant for coral. None of these scenarios matched the in situ data. (TIF) [file pone.0031192.s001.tif]
